# *SETBP1* mutations as a biomarker for myelodysplasia /myeloproliferative neoplasm overlap syndrome

**DOI:** 10.1186/s40364-017-0113-8

**Published:** 2017-12-06

**Authors:** Katherine Linder, Chaitanya Iragavarapu, Delong Liu

**Affiliations:** Department of Medicine, New York Medical College and Westchester Medical Center, Valhalla, NY 10595 USA

**Keywords:** SETBP1, Myelodysplasia, Myeloproliferative syndrome

## Abstract

Myelodysplasia (MDS) /myeloproliferative neoplasm (MPN) overlap syndrome has been described since the 2001 WHO classification as disorders that have both proliferative and dysplastic changes simultaneously. Specific disorders include chronic myelomonocytic leukemia (CMML), juvenile myelomonocytic leukemia (JMML), BCR-ABL negative atypical chronic myeloid leukemia (aCML) and unclassifiable MDS/MPN (MPN/MDS-U). Recurrent gene mutations in these conditions have been described. Among them, *SETBP1* mutations have been identified in up to 32% of aCML, 24% of JMML, 18% of CMML and 10% of MDS/MPN-U patients. The mutation hotspot lies in the amino acid residues 858–871 in the SETBP1 protein. *SETBP1* mutations in MDS/MPN overlap syndrome is associated with accelerated transformation to leukemia and poor prognosis. In this review, we summarized the latest data on the role of *SETBP1* mutations in the overlap syndrome. *SETBP1* mutations may serve as a biomarker for the diagnosis and poor prognosis of the overlap syndrome.

## Background

Myelodysplasia (MDS) /myeloproliferative neoplasm (MPN) overlap syndrome (MMOS) has been described as disorders that have both proliferative and dysplastic changes simultaneously [1–5]. Specific disorders include chronic myelomonocytic leukemia (CMML), juvenile myelomonocytic leukemia (JMML), BCR-ABL negative atypical chronic myeloid leukemia (aCML) and unclassifiable MDS/MPN (MPN/MDS-U). The overlap category of the disorders was first defined in the 2001 WHO classification of myeloid neoplasms [6] with the 2016 revision adding a fifth disorder – MDS/MPN with ringed sideroblasts and thrombocytosis (MDS/MPN-RS-T) [7]. Among MMOS, MDS/MPN-U represents about 2% of patients and usually carries a very poor prognosis with median survival of 21 months [8]. The definition of MDS/MPN-U has been updated in the 2008 version of WHO classifications [1]. A recent retrospective study validated the MDS-IPSS scoring system in MDS/MPN-U with low risk patients and delineated the natural history of this elusive malignancy [9].

There are frequent mutations in MDS, MPN, and acute myeloid leukemia (AML) [10–17]. Mutations in several pathways have been found to be useful biomarkers for diagnosis and some are druggable targets [18–22]. Ruxolitinib, enasidenib, midostaurin and several other small molecules are targeted agents in clinical applications and /or development for myeloid neoplasms [23–28]. However, no specific genetic mutations have been found for the MDS/MPN overlap syndrome. This review focused on the *SETBP1* gene mutations and summarized the clinical correlations of the common mutations in MMOS and secondary AML (sAML).

### *SETBP1* mutations

*SETBP1* was discovered in 2001 [29]. It is localized in chromosome 18q21.1. The cDNA encodes a protein with 1542 amino-acid residues. SETBP1 is a 170-kDa nuclear protein that binds to SET protein, a protein previously noted to be associated with acute undifferentiated leukemia [30].

Through whole exome sequencing of DNA from 727 patients with various myeloid malignancies, recurrent mutations of *SETBP1* were found in 52 cases (7.2%) [31]. Among them, *SETBP1* mutations have been identified in up to 32% of aCML, 24% of JMML, 18% of CMML and 10% of MDS/MPN-U patients. Somatic mutations of *SETBP1* were associated with −7/del(7q) and poor prognosis, possibly due to its gain-of-function which promotes myeloid leukemic transformation in patients with myelodysplastic syndromes (MDS) and CMML. These data were consistent with those from other reports [32–35].

## SETBP1 in AML transformation

The first clear link between SETBP1 and myeloid neoplasia was demonstrated in a patient with transformed AML [36]. The karyotyping studies performed at progression from MDS to AML in this patient revealed a t(12;18)(p13;q21) translocation leading to fusion between *ETV6* & *SETBP1*. SETBP1 protects SET from protease cleavage. Overexpression of SETBP1 was shown to increase the level of SET protein, leading to increase in the formation of the SETBP1-SET-PP2A complex [36]. *SETBP1* overexpression has been shown to confer growth advantage in hematopoietic progenitor cells, therefore promoting leukemogenic transformation. In a separate study of 386 patients with MDS, *SETBP1* mutation was found to enhance ASXL1 mutation- induced differentiation block, and played a role as critical drivers in the leukemic transformation from MDS to AML [37].

## *SETBP1* mutations in MMOS

### *SETBP1* in CMML

One study using massive parallel sequencing investigated mutational hotspots of SETBP1 in 658 patients with MDS, CMML and sAML [38]. Among the 195 CMML patients included, 12 (6.2%) were positive for point mutations in *SETBP1*, a rate higher than those in MDS (2.2%) and sAML (1.7%). The mutation hotspot affected amino acid residues 858–871 in the SETBP1 protein. Those CMML patients with *SETBP1* mutations had higher baseline WBC counts. *SETBP1* mutations were more commonly associated with ASXL1 mutations and less commonly associated with *TET2* mutations. After a median follow up of 31.1 months, *SETBP1* mutated patients were noted to have worse OS [mOS: 21 months vs. 46.4 months; HR-2.23 (95% CI 1.07-4.26, *p*-0.028)]. Interestingly, when different CD34 positive cell fractions were genotyped by single-cell genome sequencing, all 4 potential driver mutations (*ASXL1, SETBP1, ZRSR2 & SRSF2*) occurred early in the hematopoietic stem cell populations. However, *SETBP1* mutations were noted to occur after other driver mutations, and the mutant *SETBP1* clone became predominant with progression to secondary AML [38]. The prevalence of *SETBP1* mutations and their prognostic role in CMML were corroborated in other reports [39–44] (Table 1).

**Table 1:** *SETBP1* mutations in chronic myelomonocytic leukemia

Reference	Number of Patients	Mutation Prevalence	Impact on Survival
[38]	195	12 (6.2%)	Yes (*p*-0.028)
[39]	179	8 (4.5%)	Yes (*p* = 0.01)
[32]	294	21 (7.1%)	No (*p*-0.798)
[31]	152	22 (14.5%)	Yes (*p*-NR for CMML cohort)
[33]	466	21 (5%)	No (*p*-0.07)
[40]	145	26 (18%)	No (*p*-NR)
[45]	214 – Learning 260 – Validation	19 (8.9%) – Learning 24 (9.2%) – Validation	Yes (*p*-0.04)
[43]*	1364	NR	Yes (*p* < 0.001)

*NR* Not reported; * Meta-analysis

An independent prognostic scoring system for CMML, CPSS-Mol, was developed by incorporating clinical features and status of 38 commonly mutated genes in CMML [45]. The learning cohort consisted of 214 CMML patients, with 19 patients (8.9%) possessing *SETBP1* mutations. Consistent with previous reports, *SETBP1* mutations were associated with a higher WBC count (*p* < 0.001) and worse OS (HR for univariate: 2.49, *p* = 0.02; HR for multivariate: 2, *p* = 0.04). This scoring system was then validated in a cohort of 260 patients (24 patients possessed *SETBP1* mutations; 9.2%). Based on the CPSS-Mol scores, the high-risk group had an overall survival of 17 months as opposed to mOS not yet reached in the low risk group (*p* < 0.001). This new scoring system may be useful in assisting clinical decision making and future clinical trials.

### *SETBP1* in juvenile myelomonocytic leukemia

*SETBP1* mutation has also been reported in 4.8-7.2% of the patients with JMML [46, 47]. Most of the *SETBP1* mutations co-existed with RAS pathway mutations with a lower allele burden, suggesting a secondary role in leukemogenesis [46]. Interestingly, this report also demonstrated an increasing allele burden with each round of intensive chemotherapy, suggestive of association of *SETBP1* mutation with resistance to cytotoxic chemotherapy. Higher mutation rate was reported in additional studies [48, 49]. All these studies again confirmed poor prognosis in patients with *SETBP1* mutations (Table 2).

**Table 2:** *SETBP1* mutations in juvenile myelomonocytic leukemia

Reference	Number of Patients	Mutation Prevalence	Impact on Survival
[46]	92	7 (7.6%)	Yes* (*p*-NR)
[47]	42	2 (4.8%)	NR
[48]	66	23 (24.8%)	No (*p*-0.258 – Univariate) Yes (*p*-0.026 – Multivariate)
[49]	70	7 (10%)	Yes (*p*-0.03)

*NR* Not reported; * Survival analysis was done in patients harboring SETBP1 or JAK3 mutations compared to patients without either mutation

### *SETBP1* in atypical CML

aCML is one of the heterogeneous neoplasms in the group of myelodysplastic/ myeloproliferative (MDS/MPN) overlap syndrome. Unlike CML, there is no diagnostic hallmark identified in aCML [50, 51]. Due to the poor understanding of the molecular pathogenesis of this disease, diagnosis has remained elusive and the prognosis is far worse than that of CML (median survival of 37 months after diagnosis) [52]. *SETBP1* mutations was reported in 17/70 aCML patients (24%) using high throughput sequencing [34]. *SETBP1* mutation was associated with worsened survival (22 months vs. 77 months, HR for death – 2.27, *p* = 0.01) and higher WBC counts (81 × 10^3^ vs. 38.5 × 10^3^ /□L, *p* = 0.008). In a separate study that included 60 aCML patients [32], 19/60 (32%) patients were noted to have *SETBP1* mutations. *SETBP1* appears to occur with significant frequency in aCML with negative impact on prognosis. *SETBP1* mutations may be a new biomarker for aCML diagnosis and prognosis (Table 3).

**Table 3:** *SETBP1* mutations in atypical CML and unclassifiable MDS/MPN

	Reference	Number of Patients	Mutation Prevalence	Impact on Survival
Atypical CML	[34]	70	17 (24.3%)	Yes (*p*-0.01)
	[32]	60	19 (32.1%)	No (*p*-0.191)
Unclassifiable MDS/MPN	[34]	30	3 (10%)	NR
	[32]	240	20 (9.3%)	NR

*NR* Not reported

### *SETBP1* in MDS/MPN-U

In a large study of 1130 patients with myeloid neoplasms, *SETBP1* mutations were found in 20/240 (9.3%) MDS/MPN-U patients [32]. *SETBP1* mutated (mSETBP1) patients had significantly higher WBC counts and lower platelet counts and hemoglobin levels than those patients with wide-type SETBP1. Dysplastic morphology was more common in m*SETBP1* cases as compared with wild-type cases. This study also reported a significant association of *SETBP1* mutations with isochromosome i(17)(q10). The similar findings were reported by a separate group [53]. These two studies also concurred that *SETBP1* mutations were strongly associated with ASXL1 mutation. Therefore, *SETBP1* mutations can serve as a new biomarker for MDS/MPN-U (Table 3).

## Conclusion

*SETBP1* mutations are relatively common in MDS/MPN overlap syndrome. The mutation hotspot lies in the amino acid residues 858–871 in the SETBP1 protein. SETBP1 overexpression is associated with accelerated transformation to leukemia and poor prognosis. *SETBP1* mutations may serve as a biomarker for the diagnosis and poor prognosis for the overlap syndrome.

## Abbreviations

aCML: atypical chronic myeloid leukemia
CMML: Chronic myelomonocytic leukemia
JMML: Juvenile myelomonocytic leukemia
MDS: Myelodysplasia
MPN: Myeloproliferative neoplasm
MPN/MDS-U: Unclassifiable MDS/MPN
